# Selective enrichment and metagenomic analysis of three novel comammox *Nitrospira* in a urine-fed membrane bioreactor

**DOI:** 10.1038/s43705-021-00005-3

**Published:** 2021-03-25

**Authors:** Jiyun Li, Zheng-Shuang Hua, Tao Liu, Chengwen Wang, Jie Li, Ge Bai, Sebastian Lücker, Mike S. M. Jetten, Min Zheng, Jianhua Guo

**Affiliations:** 1 School of Environment, Tsinghua University, Beijing, China; 2 Department of Environmental Science and Engineering, University of Science and Technology of China, Hefei, People’s Republic of China; 3 Advanced Water Management Centre, Faculty of Engineering, Architecture and Information Technology, The University of Queensland, Brisbane, QLD Australia; 4 Department of Microbiology, IWWR, Radboud University, Nijmegen, AJ The Netherlands

**Keywords:** Microbial ecology, Freshwater ecology

## Abstract

The discovery of complete ammonia-oxidizing (comammox) *Nitrospira* has added an important new process to the microbial nitrogen cycle. While comammox *Nitrospira* have been detected in various ecosystems, only few studies have achieved their enrichment over other canonical nitrifiers. Here, we obtained a selective enrichment of comammox *Nitrospira* in a urine-fed membrane bioreactor in less than 200 days. By using 16S rRNA gene amplicon sequencing and quantitative PCR of the functional marker gene *amoA*, we observed a dominance (up to 30% relative abundance) of comammox *Nitrospira* over ammonia-oxidizing bacteria and archaea. Furthermore, the complete genomes of three new clade A comammox *Nitrospira* were recovered by metagenomics. These three strains were divergent from previously reported comammox species according to comparative genome and *amoA*-based analyses. In addition to the key genes for ammonia and nitrite oxidation, the three recovered genomes contained a complete urea utilization pathway. Our findings suggest that the urea present in the urine media played a significant role in the selective enrichment of these novel comammox *Nitrospira*, and support the diversity and versatility of their metabolism.

## Introduction

Nitrification, an essential part of the global microbial nitrogen cycle, was for a long time assumed to be a two-step process catalyzed by distinct guilds of chemolithoautotrophic microorganisms.^1^ These included ammonia-oxidizing bacteria (AOB) or archaea (AOA)^2,3^ and nitrite-oxidizing bacteria (NOB). The existence of complete ammonia oxidizers was discussed in an excellent theoretical paper in 2006,^4^ and in 2015 two experimental studies independently documented the enrichment and characterization of complete ammonia-oxidizing (comammox) *Nitrospira.*^5,6^ Intriguingly, the enriched comammox species seemed to belong to *Nitrospira* sublineage II, which was previously regarded as a canonical NOB-affiliated group.^7^ Based on the phylogenetic analysis of the gene encoding subunit A of ammonia monooxygenase (*amoA*), comammox *Nitrospira* forms two divergent sister clades, namely the comammox *Nitrospira* clades A and B.^6^ Moreover, kinetics of comammox *Nitrospira* determined with the only available pure culture revealed a high affinity for ammonia, the low maximum rate of ammonia oxidation, and high growth yield compared to canonical nitrifiers.^8^ These kinetics were recently corroborated in a highly enriched culture that was dominated by a novel comammox *Nitrospira*, which surprisingly also was partly inhibited by very low ammonium concentrations (>25 µM) in the media.^9^ These findings imply an adaptation to oligotrophic conditions and suggest that comammox *Nitrospira* can play a key role in nitrification especially in substrate-limited environments. In addition to the genes for ammonium and nitrite oxidation, a complete urea degradation pathway was also retrieved in the genomes of comammox *Nitrospira,*^5^ yet the role of urea in their metabolism and its influence on their enrichment is still not clear.

Indeed, comammox *Nitrospira* has been widely detected in various natural and engineered systems, such as drinking water production and distribution systems,^10,11,12,13^ agricultural soils,^14,15^ and wastewater treatment processes.^16–18^ In these aforementioned investigations, comammox *Nitrospira* nearly always co-occur with other nitrifiers, with whom they compete for the same substrates (mainly ammonia and oxygen). As mentioned above, their kinetic properties indicate that highly oligotrophic habitats and systems favoring slow growth are preferred niches facilitating the dominance of comammox *Nitrospira.*^8^ However, to date there are only very few enriched comammox *Nitrospira* cultures, and only one pure culture available,^5,6,8^ all of which were obtained after intensive cultivation efforts. Therefore, we asked what specific niche would be favorable for comammox *Nitrospira*? To answer this question, we monitored the nitrifying community in a membrane bioreactor (MBR) fed with real urine wastewater. It appeared that the slow ammonia release from urea hydrolysis favored the enrichment of comammox *Nitrospira* in less than 200 days.

## Materials and methods

### Bioreactor operation and sampling

A continuous-flow MBR made from Plexiglass with a working volume of 12 L was used for enrichment (Supplementary Fig. S1). The reactor was installed with a submerged hollow fiber ultrafiltration membrane module (0.02 μm pore size, Litree, China) with a total membrane surface area of 0.03 m^2^. A level control system was set up to prevent liquid overflowing. The reactor was fed with diluted real urine with Total Kjeldahl Nitrogen (TKN) concentration of 140–405 mg N L^−^^1^ (for detailed influent composition see Supplementary Table S1). Initially, the reactor was inoculated with activated sludge taken from the aeration tank of a municipal wastewater treatment plant (Tsinghua Campus Water Reuse). The pH was maintained at 6.0 ± 0.1 by adding 1 M NaOH to buffer acidification by ammonia oxidation. The airflow was controlled at 2 L min^−1^, leading to the dissolved oxygen (DO) concentration above 4 mg O_2_ L^−1^ as regularly measured by a DO probe (WTW Multi 3420). The airflow also served to wash the membrane and mix the liquid. The temperature was controlled at 22–25 °C. The initial hydraulic retention time (HRT) was 3 days and was decreased to 1.5 days on day 222. The sludge retention time (SRT) was infinite as no biomass was discharged.

The MBR was operated for 490 days. During this period, influent and effluent samples (10 mL each) were collected 1–3 times per week and used to determine the concentrations of TKN, total nitrite nitrogen (TNN = NO_2_^−^-N + HNO_2_-N), and nitrate nitrogen, according to standard methods.^19^ Mixed liquid samples (25 mL) were also taken weekly to measure mixed-liquor suspended solids (MLSS) and mixed-liquor volatile suspended solids (MLVSS).^19^ Biomass samples (10 mL) were regularly taken for qPCR and microbial community analyses (see below).

### Batch tests

In order to test urea hydrolysis and subsequent nitrification in the enrichment culture, short-term incubations were performed in a cylindrical batch reactor (8 ×18.5 cm [d × h], made from Plexiglass). 150 mL biomass was sampled from the reactor and washed three times in 1 x PBS buffer to remove any remaining nitrogen source. Subsequently, the biomass was resuspended in a 400 mL growth medium, which contained urea (about 40 mg N L^−^^1^), NaHCO_3_ (120 mg L^−^^1^), and 2 mL Hunter’s trace elements stock. Dissolved oxygen was controlled above 4 mg O_2_ L^−^^1^. Biotic and abiotic controls were performed under identical conditions with NH_4_Cl (~40 mg N L^−^^1^) instead of urea. The pH in all batch assays was maintained at 6.0 ± 0.1 by adding 1 M HCl or NaOH. According to the microbial activities during long-term operation, each batch assay lasted 6 to 8 h, and samples (5 mL) were taken every 20 to 60 min. Biomass was removed by sterile syringe filter (0.45 μm pore size, JINTENG, China), and urea, ammonium, nitrite, and nitrate concentrations were determined as described above. All experiments were performed in triplicate.

### DNA extraction

Biomass (2 mL) for DNA extraction was collected on days 0, 53, 98, 131, 161, 189, 210, 238, 266, 301, 321, 358, 378, 449, and 471. DNA was extracted using the FastDNA™ SPIN Kit for Soil (MP Biomedicals, CA, U.S.) according to the manufacturer’s protocols. DNA purity and concentration were examined using agarose gel electrophoresis and spectrophotometrically on a NanoDrop 2000 (ThermoFisher Scientific, Waltham, MA, USA).

### 16S rRNA gene amplicon sequencing and data analysis

The V4-V5 region of the 16 S rRNA gene was amplified using the universal primers 515F (5′-barcode-GTGCCAGCMGCCGCGG-3′) and 907 R (5′-CCGTCAATTCMTTTRAGTTT-3′).^20^ PCR products were purified using the AxyPrep DNA Gel Extraction Kit (Axygen Biosciences, Union City, CA, USA) according to manufacturer’s instructions and quantified using the QuantiFluor™ -ST (Promega, USA). Amplicons were pooled in equimolar concentrations and sequenced using the Illumina MiSeq PE3000 sequencer as per the manufacturer’s protocol. Amplicon sequences were demultiplexed and quality filtered using QIIME (version 1.9.1).^21^ Reads <50 bp were discarded and all remaining paired-end reads with an overlap >10 bp were assembled. UPARSE (version 7.0.1090 http://drive5.com/uparse/) was used to cluster operational units (OTUs) on a 97% similarity cut-off level, and UCHIME to identify and remove chimeric sequences. The taxonomy of each 16S rRNA gene sequence was assigned by the RDP Classifier algorithm (http://rdp.cme.msu.edu/) according to the SILVA (SSU132) 16S rRNA database using a confidence threshold of 70%.

### Quantification of various *amoA* by qPCR

To quantify the abundances of comammox *Nitrospira*, AOB and AOA in the bioreactor, qPCR targeting the functional marker gene *amoA* was performed on DNA extracted from the bioreactor at different time points. We used the specific primers Ntsp-amoA 162F/359R amplifying comammox *Nitrospira* clades A and clade B simultaneously,^12^ Arch-amoAF/amoAR targeting AOA *amoA,*^22^ and amoA-1F/amoA-2R for AOB *amoA.*^23^ Reactions were conducted on a Bori 9600plus fluorescence quantitative PCR instrument using previously reported thermal profiles (Supplementary Table S2). Triplicate PCR assays were performed the appropriately diluted samples (10–30 ng μL^−1^) and 10-fold serially diluted plasmid standards as described by Guo et al.^24^. Plasmid standards containing the different *amoA* variants were obtained by TA-cloning with subsequent plasmid DNA extraction using the Easy Pure Plasmid MiniPrep Kit (TransGen Biotech, China). Standard curves covered three to eight orders of magnitude with R^2^ greater than 0.999. The efficiency of qPCR was about 95%.

### Library construction and metagenomic sequencing

The extracted DNA was fragmented to an average size of about 400 bp using Covaris M220 (Gene Company Limited, China) for paired-end library construction. A paired-end library was constructed using NEXTFLEX Rapid DNA-Seq (Bioo Scientific, Austin, TX, USA). Adapters containing the full complement of sequencing primer hybridization sites were ligated to the blunt-end of fragments. Paired-end sequencing was performed on Illumina NovaSeq PE150 (Illumina Inc., San Diego, CA, USA) at Majorbio Bio-Pharm Technology Co., Ltd. (Shanghai, China) using NovaSeq Reagent Kits according to the manufacturer’s instructions (www.illumina.com).

### Metagenomic assembly and genome binning

Raw metagenomic sequencing reads (in PE150 mode) were trimmed and quality filtered with in-house Perl scripts as described previously.^25^ Briefly, duplicated reads caused by the PCR bias during the amplification step were dereplicated. Reads were eliminated if both paired-end reads contained >10% ambiguous bases (that is, “N”). Low-quality bases with phred values <20 at both sides were trimmed. This resulted in on average 57 million high-quality pair-ended reads for each dataset. Quality-filtered reads from each sample were assembled separately using SPAdes v3.9.0^26^ with the following parameters: –meta –k 33,55,77,99,127. Contigs >2.5 kbp were retained for later analysis. Genome binning was conducted for each sample using sequencing depth and tetranucleotide frequency. To calculate coverage, high-quality reads from all samples were mapped to the contigs using BBMap v38.85 (http://sourceforge.net/projects/bbmap/) with minimal identity set to 90%. The generated bam files were sorted using samtools v1.3.1.^27^ Then, sequencing depth was calculated using the script “jgi_summarize_bam_contig_depths” in MetaBAT.^28^ Metagenome-assembled genomes (MAGs) were obtained in MetaBAT. MAG quality, including completeness, contamination, and heterogeneity, was estimated using CheckM v1.0.12.^29^ To optimize the MAGs, emergent self-organizing maps^30^ were used to visualize the bins, and contigs with abnormal coverage or discordant tetranucleotide frequencies were removed manually. Finally, all MAGs were reassembled using SPAdes with the following parameters: –careful –k 21,33,55,77,99,127. The reads used for reassembly were recruited by mapping all high-quality reads to each MAG using BBMap with the same parameter settings as described above.

### Functional annotation of metagenomic assemblies and metagenome-assembled genomes

Gene calling was conducted for the complete metagenomic assemblies and all retrieved MAGs using Prodigal v2.6.3.^31^ For the MAGs, predicted protein-coding sequences (CDSs) were subsequently aligned to a manually curated database containing *amoCAB*, *hao,* and *nxrAB* genes collected from public database using DIAMOND v0.7.9 (*E*-values < 1e−5 ^32^) MAGs found to contain all these genes were labeled as comammox *Nitrospira* MAGs and kept for later analysis. Functional annotations were obtained by searching all CDSs in the complete metagenomic assemblies and the retrieved MAGs against the NCBI-nr, eggNOG, and KEGG databases using DIAMOND (*E*-values < 1e−5).

### Phylogenetic analysis

#### Phylogenomic tree

The taxonomic assignment of the three identified comammox *Nitrospira* MAGs was determined using GTDB-tk v0.2.2.^33^ To reveal the phylogenetic placement of these MAGs within the Nitrospirae, 296 genomes from this phylum were downloaded from the NCBI-RefSeq database. The download genomes were dereplicated using dRep v2.3.2^34^ (-con 10 -comp 80) to reduce the complexity and redundancy of the phylogenetic tree, which resulted in the removal of 166 genomes. In the remaining genomes, the three comammox *Nitrospira* MAGs and 25 genomes from phylum Thermotogae which were treated as outgroups, a set of 16 ribosomal proteins were identified using AMPHORA2.^35^ Each gene set was aligned separately using MUSCLE v3.8.31 with default parameters,^36^ and poorly aligned regions were filtered by TrimAl v1.4.rev22 (-gt 0.95 –cons 50^37^) The individual alignments of the 16 marker genes were concatenated, resulting in an alignment containing 118 species and 2665 amino acid positions. Subsequently, the best phylogenetic model LG + F + R8 was determined using ModelFinder^38^ integrated into IQ-tree v1.6.10.^39^ Finally, a phylogenetic tree was reconstructed using IQ-tree with the following options: -bb 1000 –alrt 1000. The generated tree in newick format was visualized by iTOL v3.^40^

#### *amoA* tree

Reference *amoA* sequences of AOB, AOA, and comammox *Nitrospira* were obtained from NCBI. Together with the *amoA* genes from the present study, all sequences were aligned and trimmed as described above. IQ-tree was used to generate the phylogenetic tree, with “LG + G4” determined as the best model.

#### ureABC gene tree

*ureABC* gene sequences detected in this study were extracted and used to build a database using “*hmmbuild*” command in HMMER.^41^ *ureABC* gene sequences from genomes in NCBI-RefSeq database (downloaded on July 1st, 2019) were identified by searching against the built database using AMPHORA2. The same procedures as above were conducted to construct the phylogenetic tree of concatenated *ureABC* genes, except for the sequence collection step. To reduce the complexity of the phylogenetic tree, the alignment of concatenated *ureABC* genes was clustered using CD-HIT^42^ with the following parameters: -aS 1 -c 0.8 -g 1. Only representative sequences were kept for phylogeny reconstruction, which resulted in an alignment containing 858 sequences and 1263 amino acids positions. “LG + R10” was determined as the best model and used to build the phylogenetic tree. Regarding the Nitrospirae-specific *ureABC* gene tree, *ureABC* gene sequences were recruited from the genomes as described above, but without the sequence clustering step. The final Nitrospirae-specific phylogeny of *ureABC* genes was built on an alignment containing 62 sequences and 1015 amino acid positions with “LG + F + I + G4” as the best model.

## Results

### Selective enrichment of comammox *Nitrospira* in urine-fed MBR

**Fig. 1:**
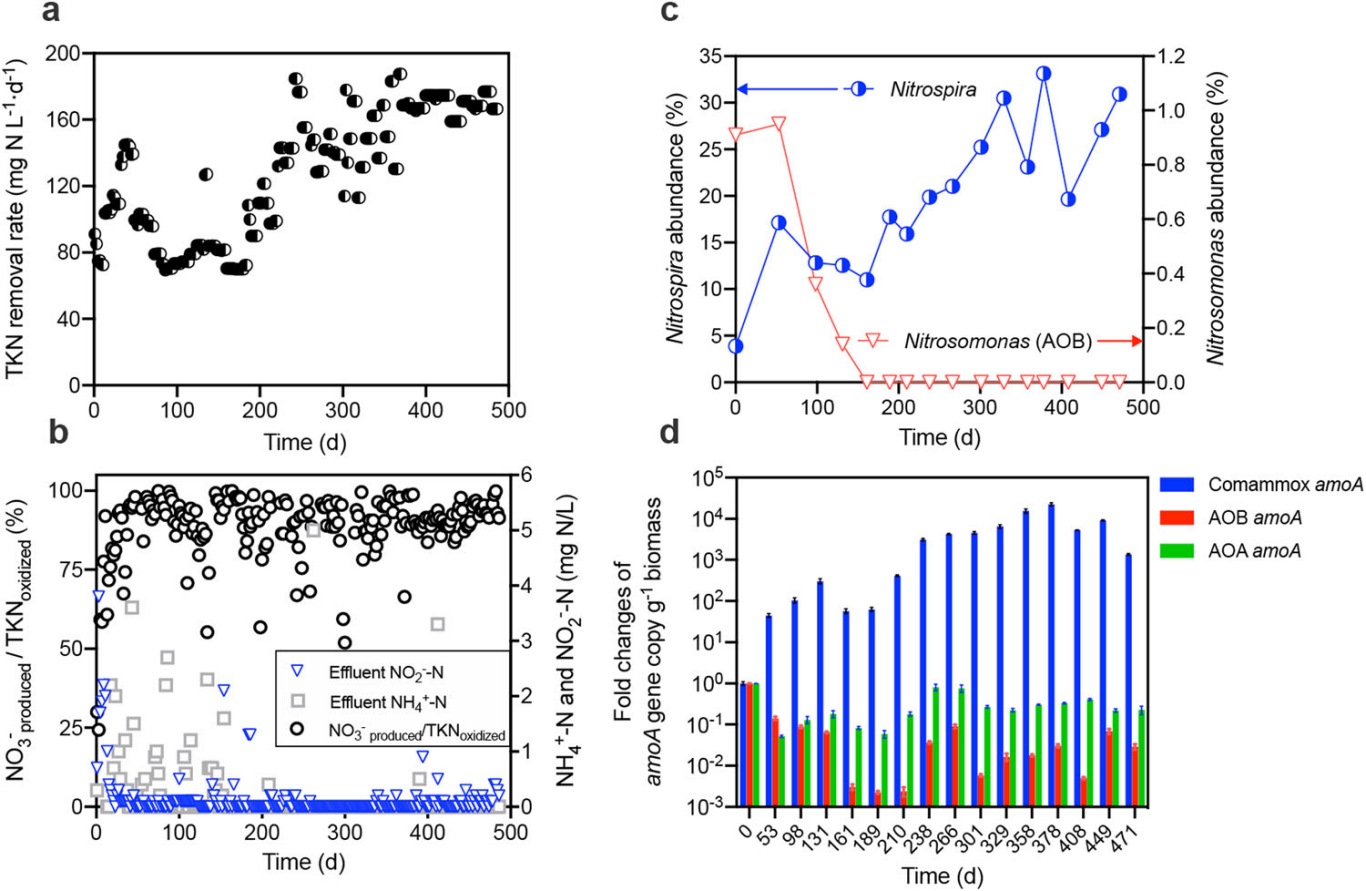
Nitrification performance and nitrifying community composition of the urine-fed MBR. **a** TKN removal rate. **b** Ammonium and nitrite conversion. **c** Variations in the relative abundance of *Nitrospira* and *Nitrosomonas* based on 16S rRNA gene amplicon sequencing. **d** qPCR-based quantification of comammox *Nitrospira*, AOB, and AOA *amoA* genes.

Initially, an MBR with a working volume of 12 L (Supplementary Fig. S1) was inoculated with nitrifying activated sludge from a municipal wastewater treatment plant with the aim to treat source-separated urine. The reactor was fed with real urine wastewater (for detailed influent compositions see Supplementary Table S1) for 490 days in total without intentional biomass removal (corresponding to an infinite SRT). Unlike any of the previous enrichments of comammox *Nitrospira,*^5,6^ oxygen in this reactor was supplied in excess to maintain the DO concentration above 4 mg O_2_ L^−^^1^. As expected, stable nitrification performance was attained rapidly at a controlled pH of 6.0 ± 0.1, in terms of ammonium conversion to nitrate, with negligible nitrite accumulation (0.1 ± 0.4 mg NO_2_^−^-N L^−^^1^). Upon a subsequent gradual decrease in HRT, the MBR achieved a maximum TKN removal rate of 188 mg N L^−^^1^ d^−^^1^ (Fig. 1a). The ratio of NO_3_^−^_produced_ to TKN_oxidized_ was nearly 100% (Fig. 1b). Batch incubations with urea (39 ± 0.3 mg N L^−^^1^) as the only nitrogen source were performed to simulate urine conversion to nitrate and confirm bona fide urea degradation (Supplementary Fig. S2). While we observed simultaneous urea degradation and nitrate increase, some ammonium still accumulated (8.4 ± 0.9 mg N L^−^^1^) in the first 3.5 h, but subsequently decreased to 0 mg N L^−^^1^ within the next 2.5 h (Supplementary Fig. S2a). While the urea degradation seems to be independent of ammonium oxidation (Supplementary Fig. S2c), there was no urea hydrolysis observed in the abiotic control (Supplementary Fig. S2d). These results suggest that the microbial community present in the MBR could use urea as an alternative ammonium source, which was converted into nitrate.

Biomass was periodically sampled for 16 S rRNA gene amplicon sequencing (Supplementary Fig. S3). Surprisingly, *Nitrosomonas* was the only detected canonical AOB with a relative abundance of 0.91% in the inoculum (day 0) but became undetectable from day 161 onwards (Fig. 1c). As no AOA-affiliated genus or NOB other than *Nitrospira* were detected in the enriched culture, we probed for the presence of comammox *Nitrospira* by using quantitative PCR (qPCR; Fig. 1d and Supplementary Table S2). During the long-term operation, the copy number of comammox *Nitrospira amoA* genes increased up to 10^4^ times, whereas a decreasing trend was observed for AOB *amoA* and AOA *amoA* genes if compared with the inoculum. After 300 days of enrichment, *Nitrospira* represented up to 33% of the total 16 S rRNA genes in the population based on amplicon sequencing (Supplementary Fig. S3). These results collectively indicated that comammox *Nitrospira* had been selectively enriched in this urine-fed MBR in less than 200 days, while AOB and AOA seemed to play only a minor role in the system.

### Metagenomic sequencing and binning

The genus *Nitrospira* consists of at least six distinct sublineages, with all known comammox organisms affiliated with sublineage II. However, within this sublineage, the comammox *Nitrospira* does not form a monophyletic clade and cannot reliably be recognized based on 16S rRNA gene amplicon sequencing.^10^ Thus, to further characterize the microbial community, we sequenced the complete metagenome of three samples (collected on day 189, 266, and 378; see “Materials and methods” section), which allowed the reconstruction of three high-quality MAGs belonging to *Nitrospira* (Supplementary Fig. S4 and Supplementary Table S3).

To identify the potential for complete nitrification in the recovered *Nitrospira*, the predicted CDSs in each MAG were probed for the presence of *amoCAB*, *hao,* and *nxrAB*, encoding the functional subunits of ammonia monooxygenase, hydroxylamine dehydrogenase, and nitrite oxidoreductase, respectively. Indeed, the complete suite of functional genes for ammonia oxidation (*amoCAB* and *hao*) were identified in all three MAGs, together with the genes for nitrite oxidation (*nxrAB*), thus confirming that these MAGs belong to comammox *Nitrospira*. The observed GC content, genome size, number of protein-coding genes, and the coding density are all in the range of previously published comammox *Nitrospira* genomes (Supplementary Table S3).^5,14,43,44^ Estimated completeness and contamination of the three *Nitrospira* MAGs were >90% and <3%, respectively, indicating their high quality and good resolution.

### Identification of three new comammox *Nitrospira*

**Fig. 2:**
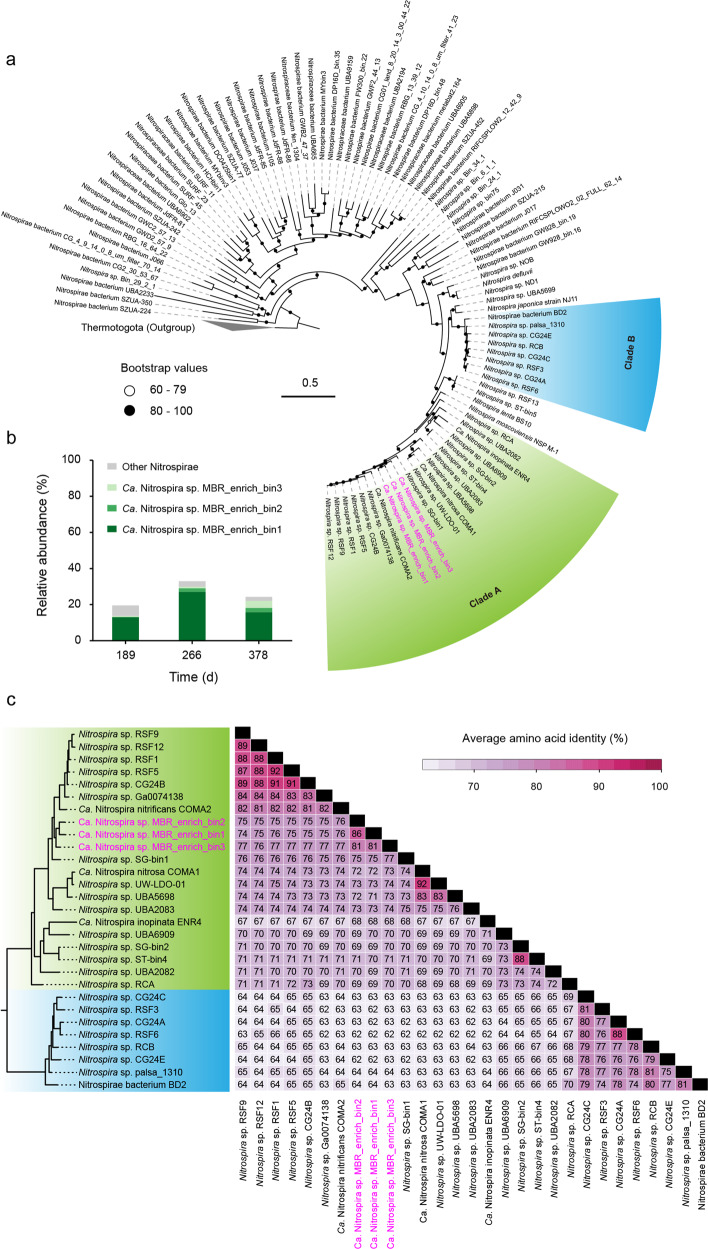
Phylogenetic affiliation and abundance of the three novel comammox *Nitrospira*. **a** Maximum-likelihood phylogenetic tree constructed on the basis of 16 concatenated ribosomal proteins. Reference genomes were downloaded from the NCBI-RefSeq database. Genomes from Thermotogae are used as outgroups. Comammox *Nitrospira* clade A and B *Nitrospira* are indicated by green and blue shading, respectively. **b** Relative abundances of comammox *Nitrospira* bins recovered from the individual metagenomes. The relative abundances were calculated as proportions of recruited clean reads mapped to a specific MAG to all clean reads. **c** Average amino acid identities (AAIs) calculated for comammox *Nitrospira* clade A and B genomes. The sub-tree on the left was extracted from the phylogenetic tree in **a**. Heatmap on the right gives the average AAI values between all 28 comammox *Nitrospira* genomes. Genomes retrieved in this study are shown in pink in **a** and **c**.

Phylogenetic analysis using a concatenated alignment of 16 conserved single-copy bacterial marker genes showed the placement of three recovered comammox *Nitrospira* MAGs within comammox *Nitrospira* clade A of *Nitrospira* sublineage II (Fig. 2a), which also includes the genomes of all previously enriched comammox *Nitrospira.*^5,6,9^ Read mapping against the assembled metagenomes indicated that the microbial community in the MBR at all sampling time points was dominated by comammox *Nitrospira*, with total relative abundances of up to 30% (Fig. 2b), which is in good agreement with the total *Nitrospira* with ≤33% observed by amplicon sequencing. In addition, the relative abundance of the only canonical nitrite-oxidizing *Nitrospira* MAG (identified based on metagenomics, Supplementary Table S4) gradually decreased from 4.0% (day 189) to 1.8% (day 378). Together with the results of 16 S rRNA gene amplicon sequencing and qPCR, this further substantiates the dominance of comammox *Nitrospira* in the MBR. Comparative genomic analyses revealed that the three comammox *Nitrospira* MAGs had average amino acid identities (AAI) ≤ 77% with 25 publicly available genomes of comammox *Nitrospira* and ≤86% with each other (Fig. 2c), indicating that they represent new species as these are well below the 95% AAI cut-off proposed for species delineation.^45^ This evolutionary divergence was also underpinned by the phylogenomic distances to their closest relatives (Fig. 2c). Consistently, the *amoA* sequences of the three comammox *Nitrospira* MAGs obtained in this study clustered within comammox *Nitrospira* clade A, confirming their affiliation with this distinct group (Supplementary Fig. S5)

### Urea degradation by comammox *Nitrospira*

**Fig. 3:**
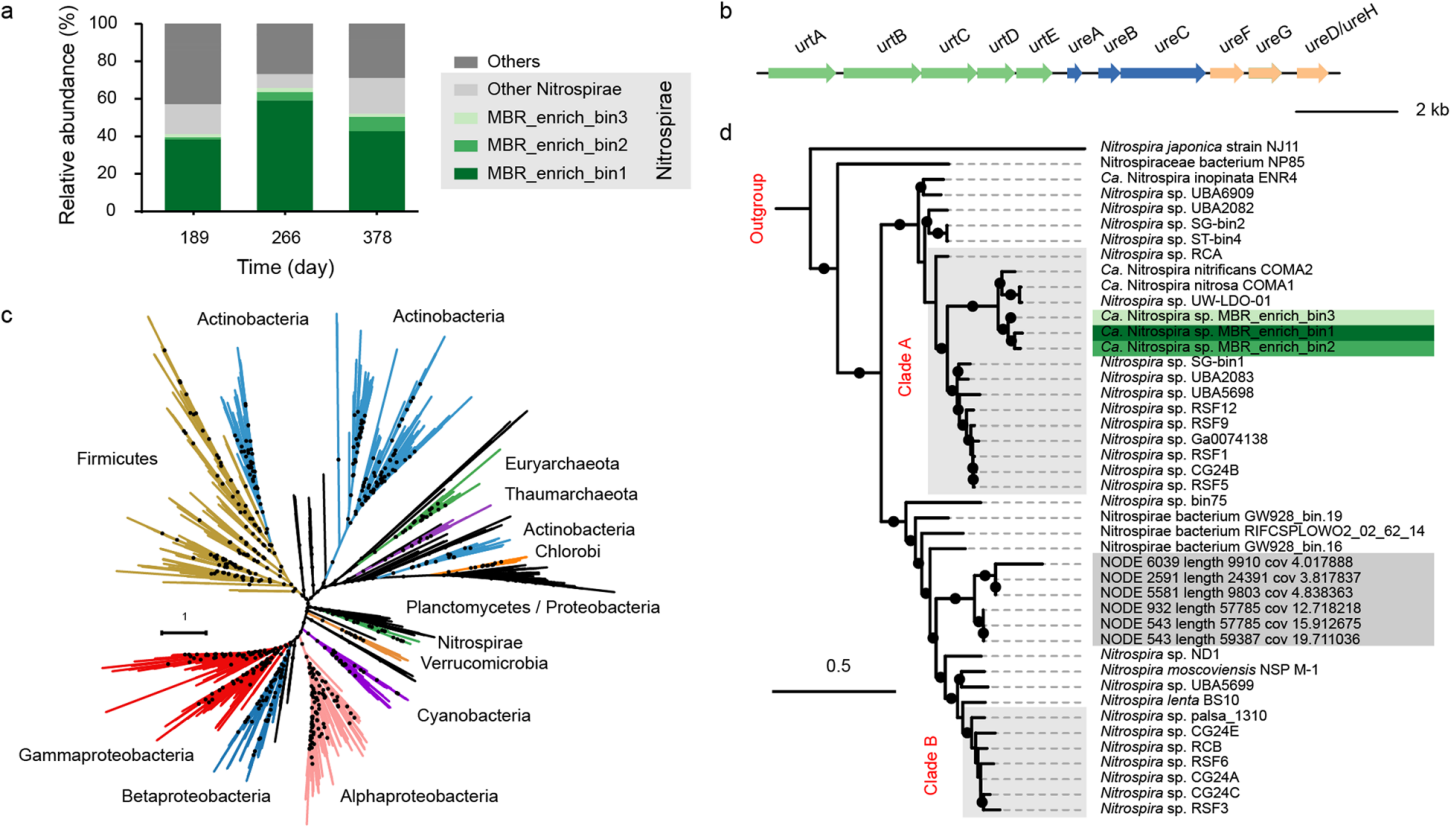
Detected *ure* operons in MBR community members. **a** Relative abundance of urease-containing microorganisms in the community. All *ureC* genes were extracted from the three metagenomes and their relative abundances were estimated by calculating the proportions of mapped reads from a specific *ureC* gene to all detected *ureC* genes. **b** Schematic representation showing the arrangement of the urease and urea transporter operons in *Nitrospira*. Arrows represent genes and indicate the transcriptional direction. Maximum-likelihood-based phylogeny of concatenated protein sequences of *ureABC* genes extracted from **c** all publicly available prokaryotic genomes and **d** all *Nitrospira* genomes. Both trees were calculated with 1000 bootstrap replications, bootstrap support ≥70% is indicated by black circles.

To infer the potential impact of urea degradation during the enrichment, the urea metabolism in the three MAGs was analyzed in detail. The full genetic complement for urea utilization was indeed present in all three species (Fig. 3a, b). The *ureABC* core genes were localized on a contig along with urease accessory genes (*ureDFGH*). Furthermore, genes for an ABC-type urea transporter (*urtABCDE*) were identified directly upstream of *ureABC*. While genes responsible for urea utilization were also detected in other community members, including bacteria affiliated with Rhodospirillales, Rhizobiales, and Actinobacteria (Supplementary Fig. S6), the comammox *Nitrospira* were most likely the key contributors for urea degradation in this system. This is underlined by the high relative abundance of comammox *Nitrospira*-derived *ureC* accounting for 41–66% of all *ureC* genes based on metagenomic read mapping. Moreover, the existence of *ureC* in other Nitrospirae indicated that canonical NOB might also contribute to urea degradation at a certain level. Remarkably, phylogenetic analysis showed that the UreABC protein sequences from the phylum Nitrospirae form a distinct deeply branching cluster within the protein family (Fig. 3c), indicating urea hydrolysis being an ancient trait of the genus *Nitrospira*. Together with the absence of sequences from non-Nitrospirae species within this sequence group, the monophyly of this lineage strongly suggests vertical inheritance of this trait from their last common ancestor. The observation that most non-*Nitrospira* members in this phylum are not possessing urease is most likely explained by frequent gene loss within these lineages. Within the genus *Nitrospira*, the *ureABC* of the three comammox *Nitrospira* obtained in this study form a monophyletic cluster, together with ureases of other clade A affiliates (Fig. 3d), which is in line with concatenated marker gene and *amoA*-based results. To identify the respective contribution of canonical NOB and comammox *Nitrospira* to urea degradation, further investigation of *ureC* expression of each genome is needed.

## Discussion

Until now, only a few comammox *Nitrospira* have successfully been enriched, as it is not yet clear how to promote their growth over other nitrifiers besides applying substrate-limited conditions favoring slow growth.^8^ Indeed, the previously obtained enrichments of comammox *Nitrospira* were initiated from biofilms in engineered systems, which fulfilled the conditions of long SRT and low substrate concentrations.^5,6^ Furthermore, low oxygen levels were recognized as a critical factor in the competition between comammox *Nitrospira* and other nitrifiers in natural and manmade systems.^46,47^ However, in this study, the enrichment of comammox *Nitrospira* was achieved at DO concentrations above 4 mg O_2_ L^−^^1^, which indicates that oxygen may not be a decisive factor to favor comammox *Nitrospira* over other nitrifiers. This observation is in line with a recent study surveying twelve wastewater treatment plants, which also showed no obvious correlation between oxygen levels and abundances of comammox *Nitrospira.*^17^ Below we discuss the potential factors that could instead have been responsible for the selective enrichment of comammox *Nitrospira* in <200 days in the present study.

Firstly, the reactor was fed in continuous mode at low pH of 6.0 ± 0.1, leading to an extremely low free ammonia concentration of ~30 nM. Meanwhile, besides biomass loss during samplings, there was no sludge discharging in the MBR, causing an infinite SRT. Presumably, both low ammonia availability and long SRT contributed to the enrichment of comammox *Nitrospira*. However, there are more natural systems with such conditions that still are dominated by other nitrifiers. For example, acidic forest soils, in which ammonia concentrations were also negligible at a pH below 5.0 and SRT can be considered as long as hundreds of days, showed one to two orders of magnitude higher *amoA*-based abundances of AOA than comammox *Nitrospira.*^10,48^ These observations indicate that long SRT and low ammonia availability are not the only decisive factors for the dominance of comammox *Nitrospira*.

**Fig. 4:**
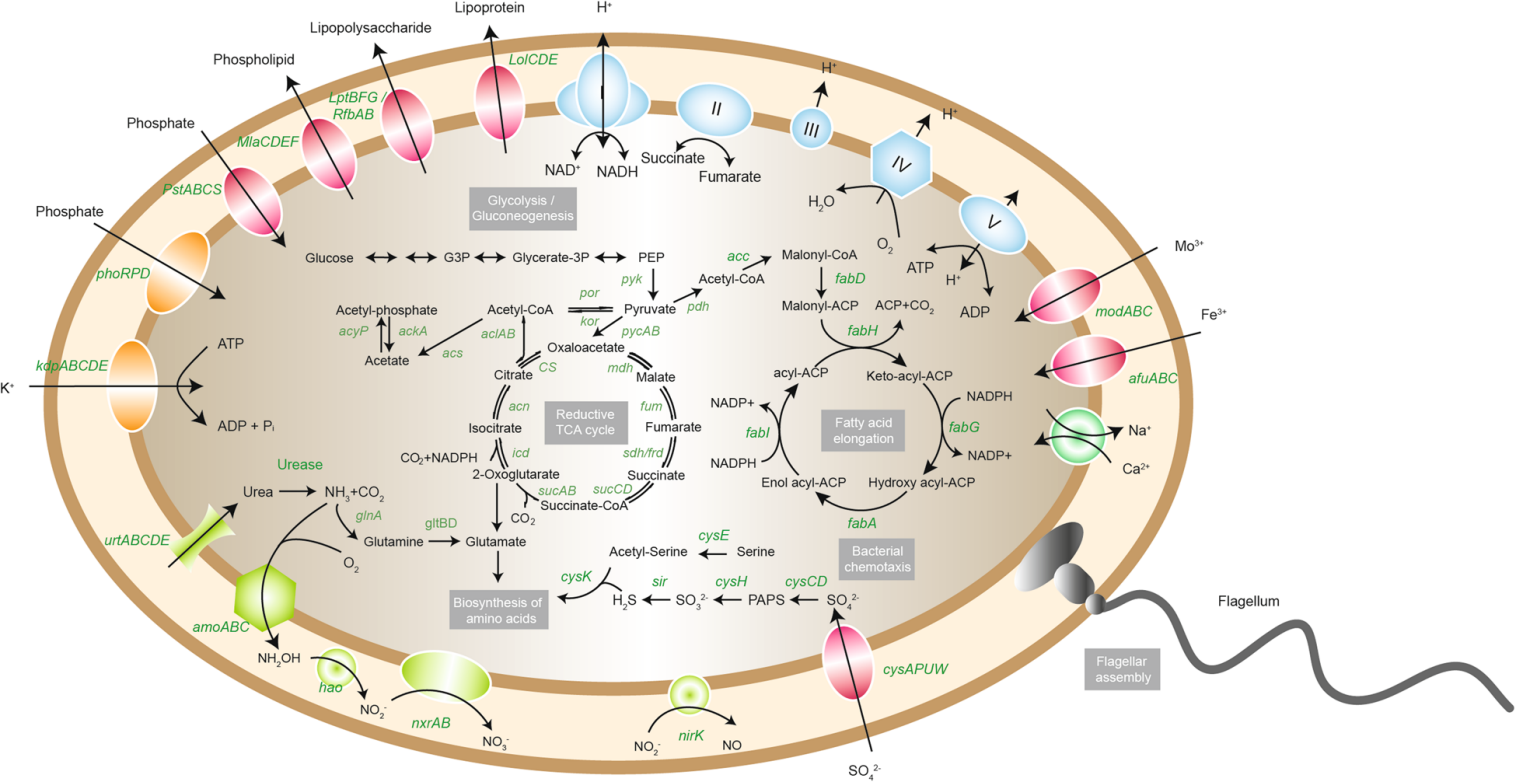
Cell metabolic diagram constructed from the MBR comammox *Nitrospira* genome annotations. Only genes relevant for the carbon, nitrogen, and energy metabolisms, and nutrient transport are shown. Solid lines indicate genes detected in the genomes of comammox *Nitrospira* retrieved in this study.

Notably, one of the critical features of the present study was urea supply in the reactor medium, which likely was another contributor for selectively enriching comammox *Nitrospira*. A complete urea utilization pathway was recovered from the enriched comammox *Nitrospira*, which agreed with previous studies.^5,43,44^ Due to the presence of the nickel-dependent urease (UreABC), accessory proteins (UreDFG), and ATP-dependent ABC-type urea transporter (UrtABCDE), urea can be enzymatically hydrolyzed to ammonia and CO_2_ by comammox *Nitrospira* (Fig. 4). Besides its role in assimilation and amino acid biosynthesis, the produced ammonia can fuel nitrification. Notably, although a small fraction of AOB and AOA can also use urea as energy and nitrogen source under ammonium-limited conditions,^49–52^ the ureolytic capability is recognized as a general feature for comammox *Nitrospira*, as the relevant genes (*ureABC*) have been detected in almost every genome of comammox *Nitrospira* retrieved up to date.^18^ Moreover, different kinetics for urea likely contributed to the selective success of comammox *Nitrospira* as well. For example, if the affinity constant of urea for comammox *Nitrospira* is indeed lower than other urea-utilizing microorganisms, they could be selectively enriched under urea-limiting conditions. This hypothesis requires experimental investigation in the future.

Urea, an organic nitrogen compound ubiquitously present in marine and freshwater systems, agricultural soil, and domestic sewage, is derived from both natural and anthropogenic sources.^53^ Although the utilization of urea has been identified in various microbial groups, the enrichment of novel comammox *Nitrospira* on this substrate is remarkable and adds to their important role in the microbial nitrogen cycle. Generally, ammonia oxidation is considered as the initial rate-limiting step of nitrification, also causing close cooperation between AOB/AOA and NOB. Recently, it has been proposed that ureolytic NOB can also initiate nitrification by cleaving urea into ammonia and CO_2_, which will then reciprocally feed the AOB/AOA.^7,54^ Evidenced by the fact that the majority of *ureC* was derived from the retrieved comammox *Nitrospira* genomes and the successful enrichment of comammox *Nitrospira* in a short time, the present study further supports that a single microorganism can carry out all reactions to convert urea to nitrate on its own,^7,49–52^ and also highlighted that urea could be a potential selective factor for the enrichment of comammox *Nitrospira*. Further investigation by culture-dependent experiments will provide more solid evidence.

## Supplementary Information

Supplementary Information

## Data Availability

All data supporting the findings of this study are available in this paper and the Supplementary Information. Sequencing data are deposited at the NCBI under the project of PRJNA637405, with raw sequence data under accession numbers SRR11928969-SRR11928971, and the draft genomes of three new comammox *Nitrospira* under the Biosample accession numbers SAMN15105088–SAMN15105090.
