# Supplementary material for: Selective enrichment and metagenomic analysis of three novel comammox Nitrospira in a urine-fed membrane bioreactor
Source: ISME Commun. 2021 Mar 25;1:7. doi: 10.1038/s43705-021-00005-3 (PMC9723585; doi:10.1038/s43705-021-00005-3)
Supplement: Supplementary file 1 — Supplementary Information [file 43705_2021_5_MOESM1_ESM.docx]

**Supplementary Information**

**Selective enrichment and metagenomic analysis of three novel comammox *Nitrospira* in a urine-fed membrane bioreactor**

**Running title:** **New comammox enriched by urine**

Jiyun Li^1,*^, Zheng-Shuang Hua^3,*^, Tao Liu^2^, Chengwen Wang^1,†^, Jie Li^2^, Ge Bai^1^, Sebastian Lücker^4^, Mike S. M. Jetten^4^, Min Zheng^2,†^, Jianhua Guo^2,†^

^1^ School of Environment, Tsinghua University, Beijing 100084, China

^2^ Advanced Water Management Centre, Faculty of Engineering, Architecture and Information Technology, The University of Queensland, St Lucia, Queensland 4072, Australia.

^3^ Department of Environmental Science and Engineering, University of Science and Technology of China, 230026 Hefei, PR China

^4^ Department of Microbiology, IWWR, Radboud University, Heyendaalseweg 135, Nijmegen, 6525, AJ, the Netherlands

*These authors contributed equally to this work.

†Corresponding authors, E-mail: [j.guo@awmc.uq.edu.au](mailto:j.guo@awmc.uq.edu.au); [wangcw@tsinghua.edu.cn](mailto:wangcw@tsinghua.edu.cn); [m.zheng@awmc.uq.edu.au](mailto:m.zheng@awmc.uq.edu.au).

**Figure S1**. Diagram of a continuous-flow membrane bioreactor system. High and low medium level control the influent pump on/off. Dosing pump was controlled by pH feedback.

**Figure S2**. The degradation of urea and ammonium as the sole energy source respectively in batch experiments. (**a.** enriched biomass + urea with oxygen; **b.** enriched biomass + ammonium with oxygen; **c.** enriched biomass + urea without oxygen; **d.** abiotic control with urea with oxygen). The error bars present the standard deviations in triplicate tests.

**Figure S3**. Overview of microbial community based on 16S rRNA gene amplicon sequencing showing all genera with relative abundance above 1.0% in at least one sample. The genera with relative abundance below 1.0% were grouped into ‘Others’.

**
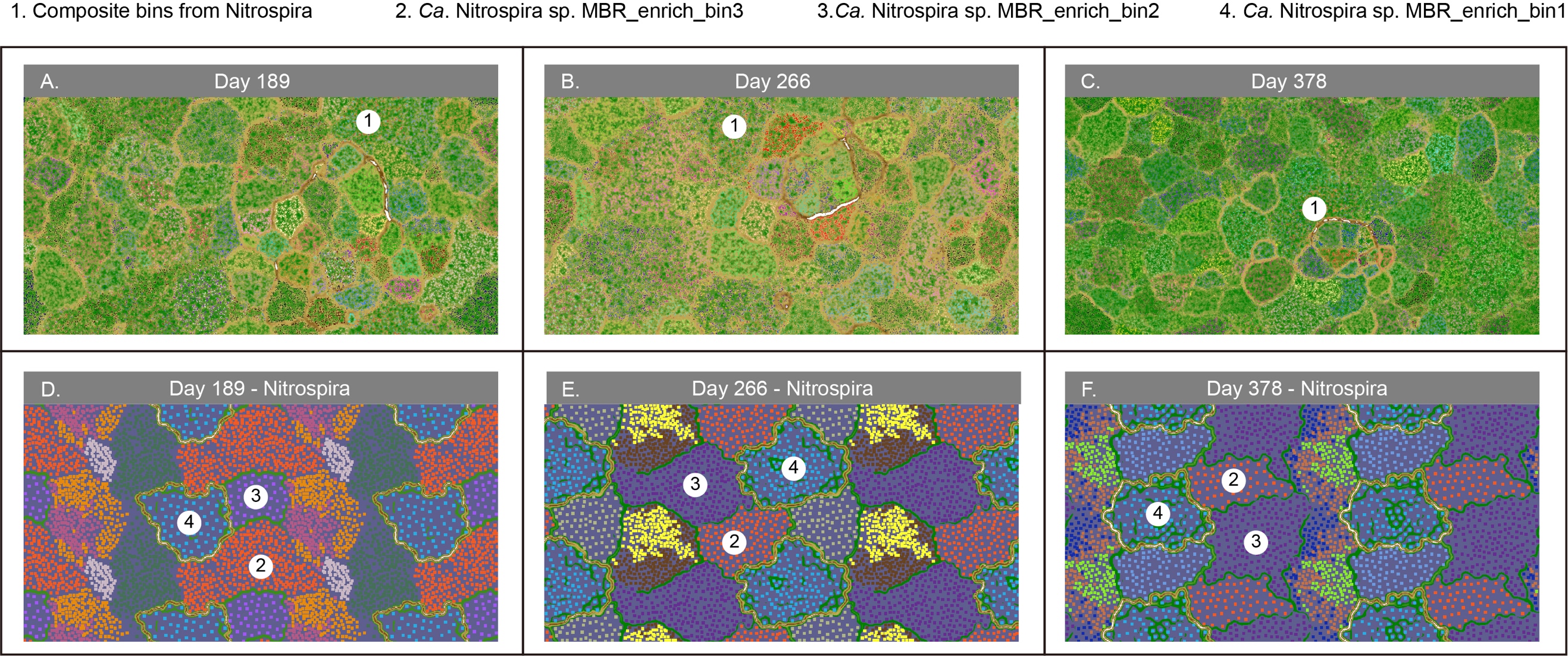
**

**Figure S4.** Emergent self-organising maps (ESOM) of metagenome contigs (≥ 5kb contigs with 5-kb window size) based on tetranucleotide frequencies. Each point is coloured based on its putative genome bin. The numbers indicate different genome assignments. Bins visualized solely based on tetranucleotide frequency (TNF) were shown in (A-C), and (D-F) by considering both TNF and sequence depth: (1) *Nitrospira* MAGs are not distinguishable simply by TNF and they formed a composite genome by mixing together, (2) *Ca*. Nitrospira sp. MBR_enrich_bin3, (3) Ca. Nitrospira sp. MBR_enrich_bin2, (4) *Ca*. Nitrospira sp. MBR_enrich_bin1.

**Figure S5.** Phylogenetic placements of *amoA* gene sequences from comammox genomes and other lineages. The maximum likelihood tree was constructed based on *amoA*, showing the phylogenetic divergence of comammox *Nitrospira* *amoA* to other superfamily *amoA* sequence (a) and within comammox clade A (b). The *amoA* gene sequences obtained in this study are highlighted by stars. Solid circles depict bootstrap support values ≥60 (based on 1000 replicates).

**Figure S6.** Schematic representation showing the arrangement of urease operons and urea transporters in four other lineages (Rhodospirillales-1, Rhizobiales, Rhodospirillales-2 and Actinobacteria). Only microbes containing both *ure* and *urt* operons are shown.

**Table S1.** Influent composition of urine-fed MBR.

| **Time (days)** | **TKN range**  **(mg N/L)** | **TKN average**  **(mg N/L)^a^** | **pH** | **TNN**  **(mg N/L)** | **NO_3_^-^**  **(mg N/L)** |
| --- | --- | --- | --- | --- | --- |
| 1-185 | 140 - 405 | 242.4 ± 63.6 | 9.0 ± 0.1 | < 3 mg/L | < 3 mg/L |
| 186-221 | 150 - 213 | 176.6 ± 20.4 |  |  |  |
| 222-490 | 139.5 - 336.5 | 211.0 ± 33.5 |  |  |  |

^a^Average ± standard deviation.

**Table S2.** Primers targeting Comammox, AOB and AOA in this study and corresponding reaction profiles

| **Specificity** | **Primer** | **Sequences (5’-3’)** | **Thermal profile** | **Reference** |
| --- | --- | --- | --- | --- |
| Comammox amoA | Ntsp-amoA 162F  Ntsp-amoA 359R | GGATTTCTGGNTSGATTGGA  WAGTTNGACCACCASTACCA | 95°C for 3 min × 1cycle; 95°C for 30s, 56°C for 30s, 72°C for 40s×35cycles. | ^1^ |
| AOB amoA | amoA-1F  amoA-2R | GGGGTTTCTACTGGTGGT  CCCCTCKGSAAAGCCTTCTTC | 95°C for 3 min × 1cycle; 95°C for 30s, 60°C for 30s, 72°C for 40s×35cycles. | ^2^ |
| AOA amoA | Arch-amoAF  Arch-amoAR | STAATGGTCTGGCTTAGACG  GCGGCCATCCATCTGTATGT | 95°C for 3 min × 1cycle; 95°C for 30s, 56°C for 30s, 72°C for 40s×35cycles. | ^3^ |

**Table S3**. Summary statistics of the *Candidatus* Nitrospira spp. MAGs reconstructed from this study.

| Bins | MBR_enrich_bin1 | MBR_enrich_bin2 | MBR_enrich_bin3 |
| --- | --- | --- | --- |
| No. of scaffolds | 65 | 97 | 40 |
| Genome size (bp) | 4,450,789 | 4,390,967 | 4,292,663 |
| GC content (%) | 56.03 | 55.83 | 55.59 |
| N50 value (bp) | 135,655 | 59,121 | 162,139 |
| No. of protein coding genes | 4,385 | 4,385 | 4,179 |
| Coding density (%) | 87.4 | 87.6 | 88.5 |
| No. of rRNAs | 3 | 2 | 2 |
| No. of tRNAs ^*^ | 51 | 41 | 44 |
| No. of genes annotated by COG ^†^ | 3018 (71%) | 3000 (72%) | 2937 (70%) |
| No. of genes annotated by KO ^†^ | 1577 (54%) | 1576 (55%) | 1564 (55%) |
| Completeness (%) ^‡^ | 94.94 | 96.76 | 95.85 |
| Contamination (%) ^‡^ | 2.73 | 2.32 | 2.78 |

^*^ tRNAs were predicted using tRNAscan-SE ^4^.

^†^ Functional annotations for all genomes were BLAST searching local databases (see Methods).

^‡^ Genome completeness and contamination were estimated using CheckM ^5^.

**Table S4**. Relative abundances of comammox *Nitrospira* and NOB *Nitrospira*.

| **Microbial groups^*^** | **Relative abundance (%)** | | |
| --- | --- | --- | --- |
|  | **Day 189** | **Day 266** | **Day 378** |
| *Ca.* Nitrospira sp. MBR_enrich_bin1 | 13.0 | 27.1 | 15.8 |
| *Ca.* Nitrospira sp. MBR_enrich_bin2 | 0.1 | 1.9 | 2.4 |
| *Ca.* Nitrospira sp. MBR_enrich_bin3 | 0.6 | 0.9 | 3.8 |
| *Ca.* Nitrospira sp. MBR_enrich_bin4 | 4 | 2.4 | 1.8 |
| Other Nitrospirae | 1.8 | 0.6 | 0.4 |

**^*^***Ca.* Nitrospira sp. MBR_enrich_bin1, *Ca.* Nitrospira sp. MBR_enrich_bin2 and *Ca.* Nitrospira sp. MBR_enrich_bin3 are COMAMMOX MAGs presented in this study, *Ca.* Nitrospira sp. MBR_enrich_bin4 is a regular NOB MAG.

**Additional references:**

1 Fowler, S. J., Palomo, A., Dechesne, A., Mines, P. D. & Smets, B. F. Comammox Nitrospira are abundant ammonia oxidizers in diverse groundwater‐fed rapid sand filter communities. *Environ Microbiol* **20**, 1002-1015 (2018).

2 Rotthauwe, J.-H., Witzel, K.-P. & Liesack, W. The ammonia monooxygenase structural gene amoA as a functional marker: molecular fine-scale analysis of natural ammonia-oxidizing populations. *Appl. Environ. Microbiol.* **63**, 4704-4712 (1997).

3 Francis, C. A., Roberts, K. J., Beman, J. M., Santoro, A. E. & Oakley, B. B. Ubiquity and diversity of ammonia-oxidizing archaea in water columns and sediments of the ocean. *Proceedings of the National Academy of Sciences* **102**, 14683-14688 (2005).

4 Chan, P. P. & Lowe, T. M. in *Gene Prediction* 1-14 (Springer, 2019).

5 Parks, D. H., Imelfort, M., Skennerton, C. T., Hugenholtz, P. & Tyson, G. W. CheckM: assessing the quality of microbial genomes recovered from isolates, single cells, and metagenomes. *Genome Res* **25**, 1043-1055 (2015).
